# Defect Analysis and Detection of Cutting Regions in CFRP Machining Using AWJM

**DOI:** 10.3390/ma12244055

**Published:** 2019-12-05

**Authors:** Pedro F. Mayuet Ares, Franck Girot Mata, Moisés Batista Ponce, Jorge Salguero Gómez

**Affiliations:** 1 Mechanical Engineering & Industrial Design Department, Faculty of Engineering, University of Cadiz, Av. Universidad de Cadiz 10, E-11519 Puerto Real-Cádiz, Spain; moises.batista@uca.es (M.B.P.); jorge.salguero@uca.es (J.S.G.); 2 Mechanical Engineering Department, Faculty of Engineering, University of the Basque Country, Plaza Ingeniero Torres Quevedo, 1, 48013 Bilbao, Spain; franck.girot@ehu.eus

**Keywords:** AWJM, waterjet, CFRP, kerf taper, surface quality

## Abstract

The use of composite materials with a polymeric matrix, concretely carbon fiber reinforced polymer, is undergoing further development owing to the maturity reached by the forming processes and their excellent relationship in terms of specific properties. This means that they can be implemented more easily in different industrial sectors at a lower cost. However, when the components manufactured demand high dimensional and geometric requirements, they must be subjected to machining processes that cause damage to the material. As a result, alternative methods to conventional machining are increasingly being proposed. In this article, the abrasive waterjet machining process is proposed because of its advantages in terms of high production rates, absence of thermal damage and respect for the environment. In this way, it was possible to select parameters (stand-off distance, traverse feed rate, and abrasive mass flow rate) that minimize the characteristic defects of the process such as taper angle or the identification of different surface quality regions in order to eliminate striations caused by jet deviation. For this purpose, taper angle and roughness evaluations were carried out in three different zones: initial or jet inlet, intermediate, and final or jet outlet. In this way, it was possible to characterize different cutting regions with scanning electronic microscopy (SEM) and to distinguish the statistical significance of the parameters and their effects on the cut through an analysis of variance (ANOVA). This analysis has made it possible to distinguish the optimal parameters for the process.

## 1. Introduction

Carbon fiber reinforced composite materials with a polymeric matrix (CFRP) are currently widely used in the industry mainly because they possess excellent specific and customizable properties, which allows CFRP characteristics that are impossible to achieve with other materials. In addition, owing to the maturity reached by the different forming technologies, their cost is increasingly approaching that of other structural materials, such as titanium or Inconel alloys. For this reason, these materials are frequently used in different industrial sectors, especially those that require incorporating weight reductions in their manufactured components [1,2].

Although CFRP applications are usually linked to the fields of transport, construction, and energy, in recent years, their use has begun to spread to other sectors such as the naval industry and in applications in consumer goods related to sport and entertainment [3].

However, in spite of all this, the CFRPs continue to generate problems after their conformation. In general, after lamination and curing, CFRPs require a machining process until reaching their final shape owing to the dimensional and geometric requirements demanded by different industries, especially the aerospace sector [4,5,6]. For this reason, it is common to perform contour milling and drilling operations on these materials with conventional machining technologies, which entails a series of issues [4,7,8].

The stiffness and abrasion of the carbon fiber together with the heat sensitivity of the matrix make the machining an industrial challenge. In general, during the machining of polymer matrix composite materials, a continuous chip is not formed, but rather a micro-chip or high hardness dust. This dust generates an abrasive environment that causes wear of the cutting tool with loss of material and initial geometry [9]. In addition, it must be removed from the cutting area to avoid damage to the cutting equipment and associated personnel. However, this is not the only problem, because the temperature generated during the process, which is increased by abrasion, causes a softening of the matrix, and even its degradation [10,11]. This can cause damage to the material and the appearance of thermal adhesion on the tool, which favors wear. This whole process is inherent to the nature of composite materials and can affect the surface integrity of the part causing damage such as delaminations, fiber fraying, degradation, or micro-cracks [12,13].

In addition, the possibility of personalization or customization of properties makes the possibilities of configuration of the material almost infinite. For the machining process, this means that the same process with the same characteristics can lead to different results.

As a consequence of the above, increasingly more alternatives to traditional machining are being proposed, where abrasive waterjet machining (AWJM) may be an alternative thanks to a relevant number of specific advantages [14,15,16,17,18,19]:

- Absence of damage due to temperature increase during cutting that could thermally damage the matrix. The water absorbs the heat generated by the impact of abrasive particles against the material.
- Tool wear is minimal compared with traditional cutting tools. In addition, this wear is independent of the material to be machined.
- Absence of high cutting forces on the workpiece and machine. Abrasive particles act as a cutting edge, generating comparatively low or negligible tangential cutting forces compared with traditional methods. Therefore, the fixation of the piece to the machine does not require complex tooling and its preparation time is reduced.
- There is no direct contact between the cutting head and the workpiece. So deformation or vibration problems are avoided.
- Although the same dimensional quality is generally not achieved as in traditional machining, the productivity rate is higher.
- Good environmental performance because no vapors or gases are generated during machining.

Although waterjet cutting is an increasingly used technology, there are difficulties in finding optimal machining parameters for CFRP that reduce the appearance of defects and improve the dimensional and geometrical quality of the machined component.

These defects produce a special negative impact on the surface integrity of the parts obtained owing to dimensional distortions of taper, a progressive increase in roughness, and striation owing mainly to the delay of the jet when it lacks kinetic energy. As a result, it is possible to find up to three possible cutting regions [20,21,22]:

- Initial damage region (IDR). The impact of the jet deforms the surface of the material by the successive impact of the particles.
- Smooth cutting region (SCR). The jet still possesses sufficient kinetic energy. In this region, the obtained roughness is reduced in comparison with the rest of the zones.
- Rough cutting region (RCR). Striations are detected at the outlet of the material owing to the loss of energy from the jet. The worst surface quality results are obtained in this area.

The defects mentioned are directly related to parameters such as traverse feed rate (TFR), stand-off distance (SOD), water pressure (WP), and abrasive mass flow rate (AMFR), as well as to material conditions and part thickness [23,24]. Table 1 shows the main technological parameters and their influence on the defects mentioned.

There are publications that use similar materials and parameters that study defects and the dimensional and geometrical quality of the tests performed. However, in the study of microgeometry or surface quality, where there are several criteria in relation to possible areas of roughness, it has been detected that there are no publications that identify the influence of parameters in different regions.

In this article, the main objective is to study the influence of the main technological parameters in AWJM in order to obtain cutting conditions that minimize the appearance of defects by analyzing each cutting region. Likewise, the detection of different cutting regions characteristic of the process will be analyzed.

For this purpose, straight cuts using AWJM technology were performed to determine the influence of the main cutting parameters on CFRP composite material in contour machining operations. Specifically, roughness was measured in three differentiated zones in order to detect the different regions (IDR, SCR, and SCR) that can be produced during cutting, trying to distinguish the border of each of them through statistical analysis and scanning electronic microscopy (SEM).

## 2. Materials and Methods

### 2.1. Experimental Development

For the experimental development, a CFRP composite plate formed with 20 layers of prepreg 0.2 ± 0.1 mm thickness (Carbures, Cádiz, Spain) was used to form a 4 mm final plate. The main characteristics of the material are shown in Table 2.

**Figure 1:**
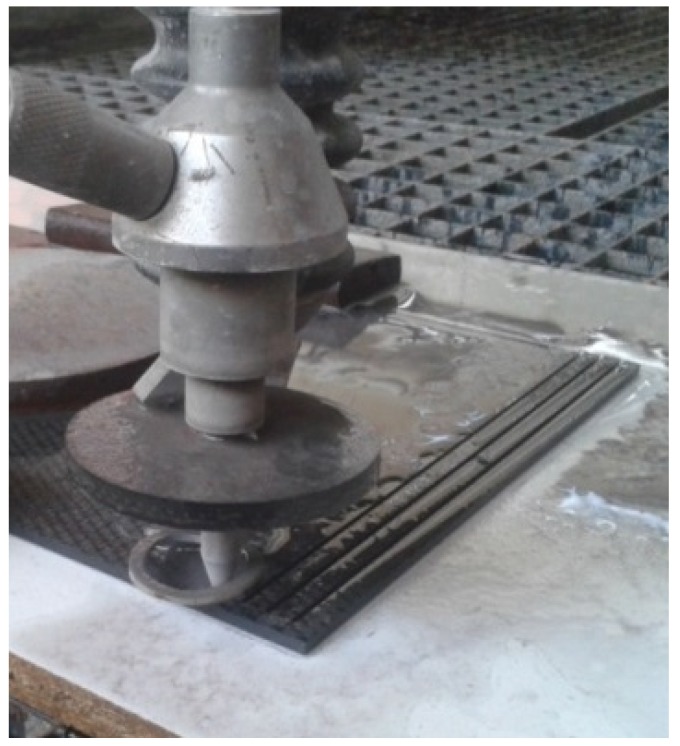
Mecanumeric MECAJET performing carbon fiber reinforced composite material with a polymeric matrix (CFRP) cutting.

The machining process consisted of the generation of 24 straight cuts, combining the parameter levels shown in Table 3. The length of each test was 180 mm in order to guarantee stable cutting conditions. For this purpose, Mecanumeric MECAJET (Mecanumeric, Marssac-sur-Tarn, France) waterjet machine was employed for cutting the composite, as shown in Figure 1. Table 4 shows the cutting parameters that were kept constant, where the pressure used in the tests is the maximum allowed by the equipment.

### 2.2. Test Evaluation

**Figure 2:**
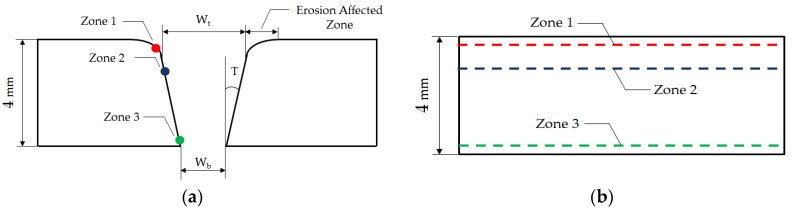
Defects diagram and evaluation zones of the cutting process with (**a**) parameters for measuring taper; (**b**) roughness measurement zones.

The variables evaluated were the angle of taper T and the roughness through the parameter Ra (arithmetic average of the roughness profile) distributed in three zones: Zone 1, Zone 2, and Zone 3, as shown in Figure 2. The distribution of roughness measurements was carried out as follows:

- Zone 1. At the entrance of the cut in the erosion affected zone (EAZ). This zone can be identified with IDR.
- Zone 2. Following the EAZ. Specifically, 1 mm from the entrance zone of the jet. This zone can be indentified with SCR.
- Zone 3. The measurement was made at the outlet of the jet. Specifically, to 3.5 mm of the entrance of the jet. This zone can be identified with RCR and is related to the jet delay effect.

For the evaluation of straight cuts, optical evaluation of the machined material was performed by means of the scanning electronic microscope (SEM) technique. For that, a Hitachi SU 1510 (Hitachi, Tokio, Japan) microscope was used for SEM inspection. With respect to roughness measurements, a station Mahr Pertometer Concept PGK 120 (Mahr, Göttingen, Germany) was used.

**Figure 3:**
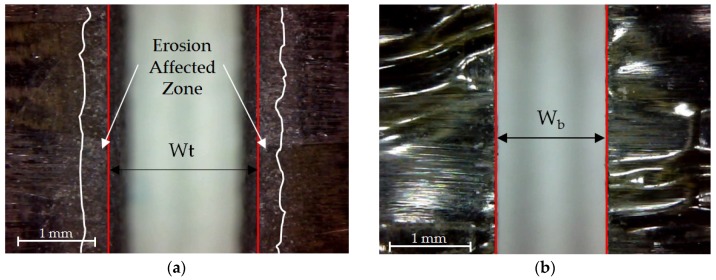
Difference between (**a**) cutting at material inlet cut at the entrance of the material showing the width at the top along with the erosion-affected zone; (**b**) cutting at material outlet showing the width at the bottom.

For T calculation, the expression of Equation (1) was used, where T is the conical angle of the slot, W_t_ is the width of the slot at the jet inlet, W_b_ is the slot width at the jet outlet, and t is the thickness of the sample. For the measurement of W_t_ and W_b_, the software ImageJ was used. A previous calibration was carried out to obtain a pixel/mm ratio that allows to establish a correct measurement directly on the image [32,33]. In addition, it is important to note that the area affected by erosion caused by the loss of jet coherence at the jet inlet was differentiated, as shown in Figure 3.

(1) $$T=tan^{-1}\left(\frac{Wt-Wb}{2t}\right)$$

### 2.3. Statistical Analysis

The analysis of results was carried out by comparing combinations of parameters through interaction graphs. In this phase, an attempt was made to identify growth or decrease trends between parameters in relation to the variables studied.

In a second phase, the objective was to quantify the influence of the parameters on the cutting process, by means of an analysis of variance (ANOVA) with a 95% confidence interval. Thus, the F-value and the *p*-value were analyzed to measure the evidence against the null hypothesis and the significance (S) of each parameter.

Finally, contour graphs were represented, emphasizing the discussion of parameters whose significance was demonstrated by ANOVA. The analysis was carried using Minitab statistical software.

## 3. Results and Discussion

### 3.1. Global Analysis of Results

The results obtained after the measurement process are shown in Table 5. The interaction graphs obtained for each of the four variables are described below in order to visualize trends between the levels of parameters used.

**Figure 4:**
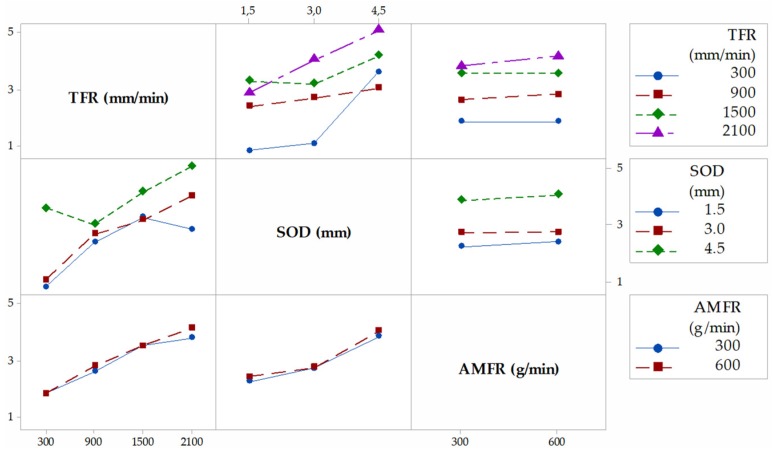
Interaction graph for T. TFR, traverse feed rate; SOD, stand-off distance; AMFR, abrasive mass flow rate.

Figure 4 shows the interaction graph for the variable T, where the different marginal relationships between parameters are observed. In a first observation, it is shown how the highest results of T are obtained when the levels of the parameters SOD and TFR are 4.5 mm and 2100 mm/min, respectively. This is in good agreement with the works of [29,33]. Thus, with a high value of SOD, the jet loses coherence and increases T. An increase of the TGF means that the jet remains for less time impacting the same area of the piece with the consequent loss of its penetration capacity. The combination of these two factors with high levels favors the formation of the defect, and vice versa. AMFR does not have a defined influence for both levels. Indeed, for 300 g/min and 600 g/min, the taper does not seem to increase considerably, although it is true that, for the highest level, there seems to be a slight increase in taper [34]. This could be because of an excess of particles directed by the jet that impact each other before reaching the cutting surface, so their size varies owing to fractures between particles, as well as their kinetic energy before impact.

**Figure 5:**
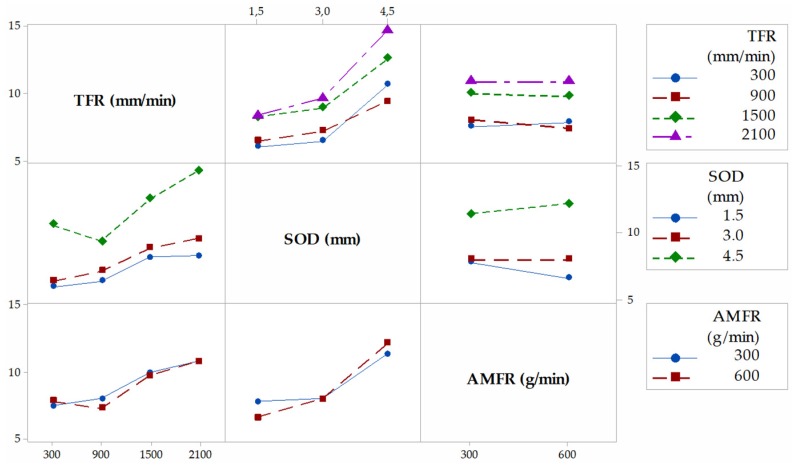
Interaction graph for Zone 1.

**Figure 6:**
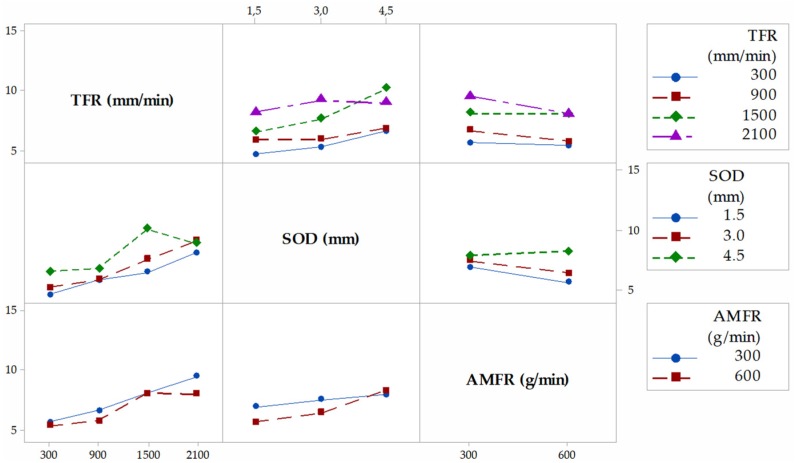
Interaction graph for Zone 2.

**Figure 7:**
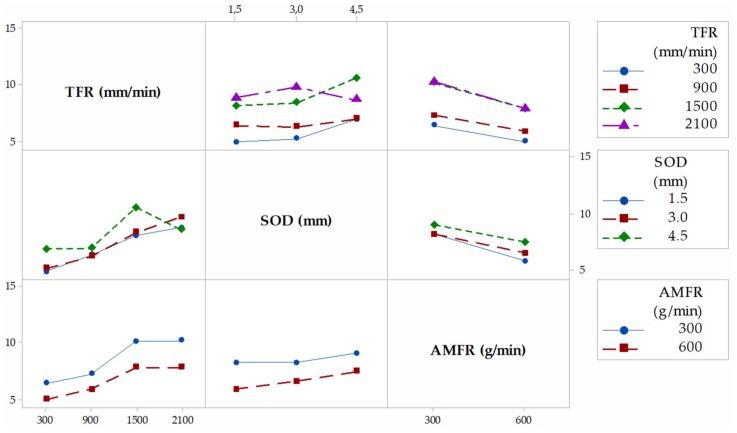
Interaction graph for Zone 3.

Figure 5, Figure 6 and Figure 7 shown the interaction graphs for the roughness values obtained in Zone 1, Zone 2, and Zone 3, respectively.

**Figure 8:**
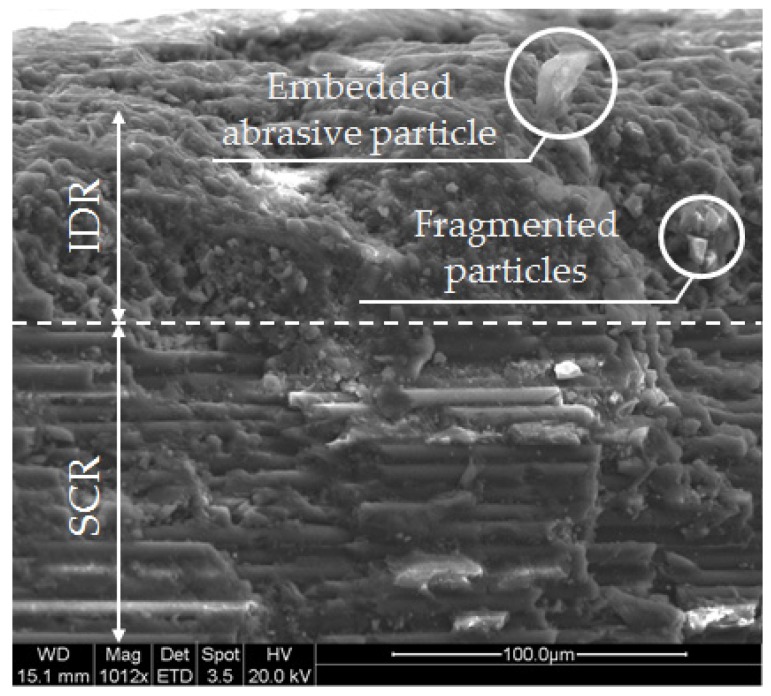
Border between the initial damage region (IDR) and smooth cutting region (SCR) formation. Particles embedded in the surface of the material and another group fragmented as a result of the cutting process are observed. Test 12. TFR = 900 mm/min; SOD = 4.5 mm; AMFR = 600 g/min.

Zone 1. This zone contains the highest roughness measurements, where some Ra values reach 15 µm. This shows the existence of an initial damage region at the input of the compound produced by the random impact of particles and consequent deformation of the material at the beginning of the cut. Thus, Figure 5 shows how SOD and TFR are the parameters that influence the process, increasing the roughness as higher values are selected [22]. An example of this can be the comparison of test 1 (TFR = 300 mm/min, SOD = 1.5 mm, and AMFR = 300 g/min) and test 19 (TFR = 2100 mm/min, SOD = 1.5 mm, and AMFR = 300 g/min), where the roughness experiences an increase from 5.87 µm to 8.71 µm. Finally, the AMFR parameter does not seem to show a clear influence during the machining process, because the values in this area do not vary considerably [35]. Figure 8 shows an image taken by SEM distinguishing the transition zone from IDR to SCR. A first observation shows that the IDR zone is completely damaged by the impact of particles, while the SCR zone maintains certain integrity in terms of the structure of the material. Specifically, the particles that erode the surface of the material produce the area affected by erosion by damaging the matrix. Subsequently, the fibers without a matrix are broken in the area where the jet penetrates, generating the mechanized groove. In this process, some particles are embedded in the material in the upper zone as a result of the impact on the material or collisions of particles with each other before impacting [36,37].

Zone 2. The values obtained in this area show a decrease in roughness with respect to previous values. This is because of two different factors: this zone does not show deformation caused by the random impact of particles as in the previous case and the jet still contains sufficient kinetic energy because the measurement is made in an area close to the entrance of the jet. This is in good agreement with the search for a SCR zone that shows a cut without the appearance of defects or striations [21]. Figure 8 shows how the SOD parameter reaches the maximum roughness values, around 10 µm, for the most unfavorable cutting conditions for the highest levels of SOD and TFR. It should be noted that, once again, AMFR does not seem to show a clear trend in roughness variation when values of 300 g/min or 600 g/min are selected, although it is true that, for the latter value, the roughness seems to decrease when TFR presents levels of 300 mm/min and 900 mm/min. 

Zone 3. This zone presents similar values to those obtained in the previous zone, although the behavior of the parameters of roughness varies. The SOD parameter does not seem to have a clear influence on the process in this case, as the results are kept within the stability when TFR and AMFR are fixed, as shown in Figure 6. In addition, it is observed that, although TFR continues to maintain a growth in roughness for the highest feed rate values, the AMFR parameter shows an even greater change in its behavior. Thus, an increase in the abrasive level of 600 g/min shows a clear decrease in roughness compared with 300 g/min. This indicates that the jet requires more abrasiveness to maintain low roughness values in this zone. This phenomenon is shown especially at high feed rates, where roughness decreases by up to 25%. Therefore, although the definitive values show values similar to those in Zone 2, a change in the influence of parameters is observed, which will be studied in depth with the ANOVA.

**Figure 9:**
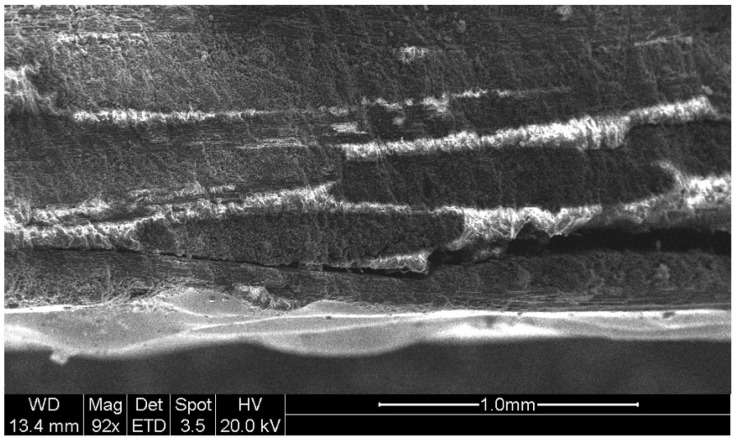
Absence of striation or waviness that visibly differentiates the difference between the smooth cutting region (SCR) and rough CR (RCR). Test 22. TFR = 2100 mm/min; SOD = 3.0 mm; AMFR = 300 g/min.

Figure 9 shows an SEM image of a test carried out with the highest level of speed and the lowest level of abrasiveness. Precisely, these levels would favor the appearance of RCR, but as can be seen, the zone appears free of striations. In addition, at the outlet of the jet, there is a delamination formed by the combination of cutting parameters. Specifically, the pressure combined with the expansion of the jet in the material produces stresses that can prevent the layers of the composite from remaining together, causing their separation and the formation of cracks. As the cutting process continues, the abrasive particles lodge in the cracks and consequently expand, forming delaminations [38,39,40].

### 3.2. ANOVA

This section analyses the degree of influence of each parameter by zone. For this purpose, Table 6 shows the F-value and *p*-value for T, Zone 1, Zone 2, and Zone 3.

**Figure 10:**
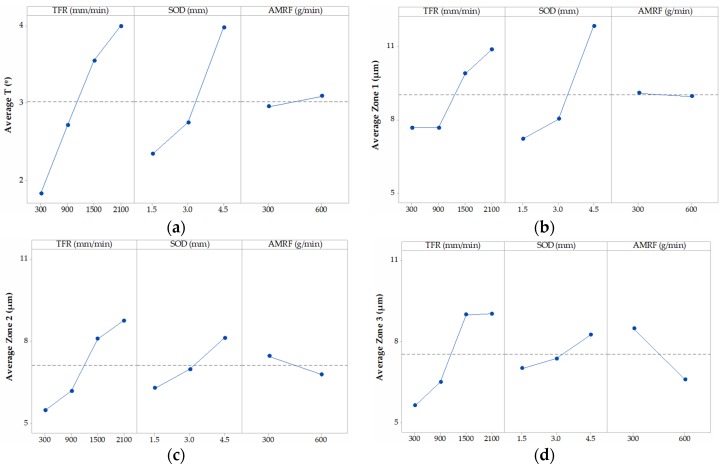
Parameter interaction graph for the response variable for (**a**) T; (**b**) Zone 1; (**c**) Zone 2; and (**d**) Zone 3.

In Figure 10a, the main effects graph for variable T is shown. The data reflect that the parameters that have significance in the process are SOD and TFT, with F values of 21.45 and 20.18, respectively. Specifically, the SOD parameter seems to have a more definite influence on the formation of the defect when the selected level increases from 3.0 mm to 4.5 mm, as reflected in the slope of the graph, while the same increase in slope for TFR was detected in the initial levels from 300 mm/min to 900 mm/min [26]. AMRF does not seem to have a definite influence on the formation of T.

As for the formation of possible roughness zones that can be detected, Figure 10b–d show the main effect graphs for Zone 1, Zone 2, and Zone 3. The degree of influence of each parameter on the three variables is described below [31,41,42,43]:

- Zone 1. The SOD and TFR parameters show the greatest degree of influence, although it is true that SOD shows the highest F-value, with a result of 52.35. This shows the importance of this parameter on the IDR zone owing to its effect on the coherence of the jet. This is reflected in Figure 10b with an increase in the mean value obtained as the distance increases. As for TFR, it was determined that it also has a degree of significance over the variable, but its influence is less. Thus, the figure shows that the parameter tends to increase roughness as speed increases, as reflected by the slope of Figure 10b for levels of 1500 mm/min and 2100 mm/min. The AMRF parameter has a reduced influence and has no significance on the process.
- Zone 2. In this case, the parameters that show significance with the cutting process are repeated: TFR and SOD. However, with a different level of influence, as reflected in Table 5. Thus, the F-value decreases from 52.35 in Zone 1 to 8.36 in Zone 2 for SOD, and increases from 16.82 in Zone 1 to 17.65 in Zone 2 for TFR. These data reflect that, while TFR maintains a similar degree of influence, SOD suffers a severe decrease as the jet penetrates the machined piece. These values are reflected in Figure 10c, where the highest roughness values are because of the influence of TFR for levels from 1500 mm/min and 2100 mm/min. In this case, AMFR does not seem to have a determining influence on the formation of the defect. However, the increase of the F-value from 0.11 in Zone 1 to 3.26 in Zone 2 should be highlighted. This is reflected in the slope of the figure and in the decrease of the *p*-value in Table 5 from 0.744 obtained in the previous zone to 0.089.
- Zone 3. In the last zone of roughness measurement, the parameters that show significance with the process are AMFR and TFR. In the first case, AMFR describes a strong relationship, showing the highest F-value with a result of 31.45, as described in Table 5. This is reflected in Figure 10d comparing the slope of the graphs obtained in the three zones, where it is observed as the slope that joins the average values of both levels of abrasive increases. This suggests that the appearance of the RCR zone is formed in machined samples with low levels of abrasiveness. As for TFR, the increase of the F-value to 26.62 illustrates what has been said so far. The degree of influence continues to increase with respect to the two previous areas and a considerable increase in roughness is observed for levels with high speed. The greatest increase in roughness occurs in the increase range of 300 mm/min to 900 mm/min. Finally, the influence of the SOD parameter decreases until it loses significance in the process, as shown in Table 6, and its reduced slope in Figure 10c with respect to the previous zones.

Therefore, everything seems to indicate that, as described, the analysis by zones reveals that, although it is possible to establish that there are two identified zones of roughness, IDR and SCR, the measurements carried out in Zone 3 reveal a change in the importance of parameters, revealing that TFR, and above all, AMFR, acquire greater significance to the detriment of SOD. Therefore, it could be considered that, with a larger plate thickness, it would have been possible to detect the presence of striations [30].

### 3.3. Analysis of Contour Graphs for Significant Parameters

This section shows the contour graphics as a function of the significant variables. The aim of this analysis is to evaluate the most favourable cutting conditions within the range of parameters studied.

#### 3.3.1. Taper Analysis

**Figure 11:**
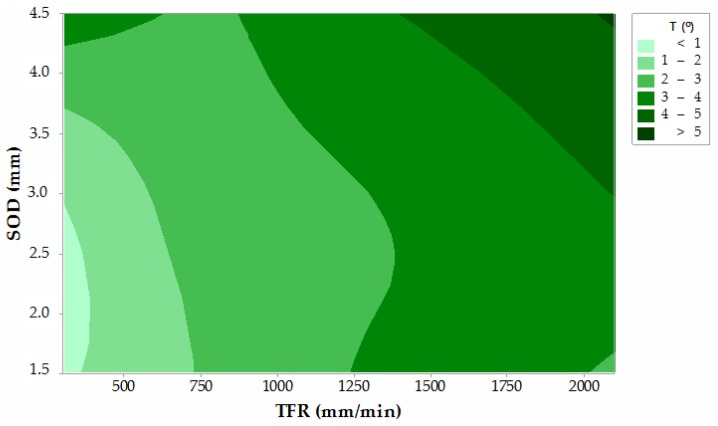
Contour graph of T as a function of parameters with significance: TFR and SOD.

Figure 11 shows the contour graph for T as a function of TFR and SOD. In this case, the AMFR parameter is irrelevant. The data obtained reflect how the cutting parameters where the taper decreases are in the range of feed rates and reduced distances. In this way, the jet can remain longer eroding the material reducing the loss of coherence of the abrasive jet. These parameters coincide with the levels of TFR = 300 mm/min and SOD = 1.5 mm, although it is reflected in the graph that distances up to 3 mm can maintain the efficiency of the cut, minimizing the growth T. In this case, the conicity can be maintained in values lower than 1°.

#### 3.3.2. Roughness Analysis

**Figure 12:**
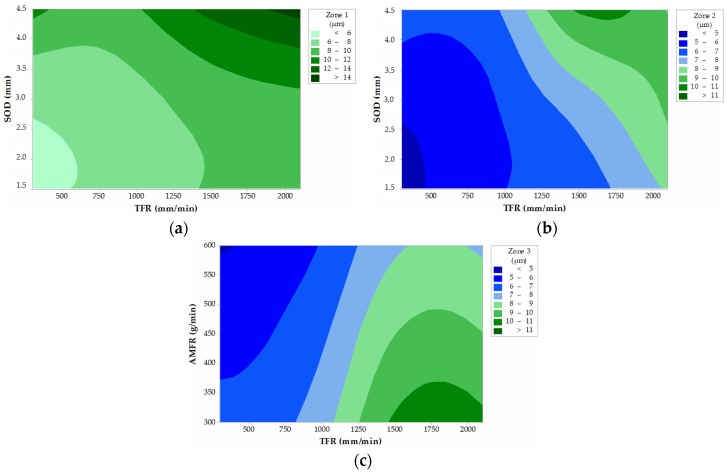
Roughness contour graphs as a function of significant parameters: (**a**) Zone 1: TFR and SOD; (**b**) Zone 2: TFR and SOD; and (**c**) Zone 3: TFR and AMFR.

As in the previous case, Figure 12 shows the contour graphs for Zone 1, Zone 2, and Zone 3. In the first two cases, the parameters with significance in the process are TFR and SOD, while at the output of the jet, the AMRF parameter is the most relevant with TFR.

It is shown that, globally, the results where roughness decreases occur when TFR and SOD are reduced, while the AMFR parameter increases. This is in good agreement with the works of [18,33], although it should be noted that, with this process, it has not been possible to obtain roughness levels lower than 4 µm. In addition, it is necessary to mention that Zone 1, corresponding to IDR, contains the highest roughness results, as has been revealed. For Zone 2 and Zone 3, the most favorable results are less than 5 µm. Although, it is to be expected that in Zone 3, identified as the possible RCR, the results are higher, and the comment has been made that the thickness of the specimens has a considerable influence. However, the ANOVA reveals that there is a change of trend in the significance of the variables that has allowed to reveal that SOD has no influence in Zone 3, highlighting the importance of AMFR in the cutting process.

## 4. Conclusions

A study was carried out on the influence of the main technological parameters on abrasive water jet involved in the formation of defects and roughness zones on CFRP material. From the study carried out in this article, the following conclusions are drawn:

- The values evaluated for T in the experiment varied between 1° and 5°. The data showed that the highest values of T are obtained when TFR and SOD reach 2100 mm/min and 4.5 mm, respectively.
- The initial assessment of surface quality clearly shows that there are at least two well-defined roughness zones: IDR and SCR. The boundary between both zones was verified using SEM techniques. Measurements taken in Zone 1 showed roughness data above 15 μm for elevated SOD and TFR, while in Zone 2, values about 10 µm were obtanined under the same conditions. This results in approximately 35% lower roughness between zones.
- Initially, the roughness analysis carried out in Zone 3 did not reveal any changes with respect to the values measured in Zone 2. However, the ANOVA of the data showed a significant change in the influence of parameters. In this sense, AMFR acquires great significance in the process, revealing its importance in the formation of RCR. Thus, in Zone 2 or SCR, the significant parameters were TGF (F-value = 17.35) and SOD (F-value = 8.36), while in Zone 3, the significant parameters were AMFR (F-value = 31.45) and TGF (F-value = 26.32).

Once the analysis of the data was completed, contour graphs with the significant parameters for each variable were represented in order to record the parameters that minimize taper and roughness defects. From these results, the following conclusions are also drawn:

- Parameters that minimize the effect of taper during cutting are achieved with SOD values between 1.5 and 3.0 mm and TFR values lower than 350 mm/min.
- The roughness data for IDR can be considerably reduced with SOD values lower than 2.0 mm and TFR values lower than 500 mm/min.
- In Zone 2 or SCR the lowest roughness data of all cutting regions are obtained with parameters similar to those recommended in the IDR.
- Roughness data in Zone 3 or RCR can be reduced when using low TFR values combined with high abrasive rates. In this experimental case, for values higher than 550 gr/min.

On the above basis, the experiment carried out shows that, in order to obtain the minimum T values and high surface quality during the cutting process of CFRP composite specimens, SOD values between 1.5 and 2.0 mm, reduced TFR values around 300 mm/min, and high AMFR values must be selected. 

Finally, in order to avoid the appearance of cracks and delaminations, it is recommended to establish a compromise between the pressure used and the thickness of the composite plate. Thus, it is established as a line of future works to obtain cuts with the absence of delaminations under the considerations made throughout this article.

## Figures and Tables

**Table 1:** Influence of main abrasive waterjet machining (AWJM) parameters on cutting quality. TFR, traverse feed rate; SOD, stand-off distance; WP, water pressure; AMFR, abrasive mass flow rate; RCR, rough cutting region; SCR, smooth CR.

Parameter	Influence on Taper	Influence on Roughness
TFR	An increase in traverse feed rate means that the jet remains on the cutting surface for less time. This results in less particle impact, making the machined slot narrower [25].	An increase in traverse feed rate causes an increase in roughness because there are less abrasive particles per unit area acting on the material. This effect accentuates the formation of RCR [26].
SOD	As SOD increases, the jet loses greater coherence, which translates into an increase in the diameter of the jet and therefore of T. This effect is particularly significant at the entry of the cut [27].	A larger diameter of the jet causes an initial damage region that is more deformed by the impact of particles and, therefore, produces an increase in roughness in this region. SOD loses influence as the jet penetrates the composite [28].
WP	An increase in pressure leads to an increase in the kinetic energy of the jet causing greater erosion in the material, leaving a smoother surface. The roughness worsens as the jet loses energy, affecting the formation of SCR [29].	An increase in pressure produces an increase in taper. This effect is more significant when the thickness of the material increases [30].
AMFR	There is no direct correlation between abrasive mass flow variation and T. Normally, increased abrasive flow results in a non-significant increase in taper angle [29].	An increase in abrasive mass flow produces a greater presence of abrasive particles, which avoids the appearance of SCR during cutting. As a consequence, it generally generates a good surface quality at the exit of the cut. However, it can also cause collisions between particles, resulting in a decrease in the effectiveness of the cut [31].

**Table 2:** CFRP plate features.

Type of Material	Composition	Production Method	Technical Specification
Layers of carbon fiber with epoxy resin matrix and a symmetrical stacking sequence of (0/90)	Intermediate module fiber (66%) and epoxy resin (34%)	Prepreg and autoclaved at 458° ± 5° at a pressure of 0.69 MPa	AIMS-05-01-002

**Table 3:** Cutting parameters selected for the tests.

Parameter	Levels			
TFR (mm/min)	300	900	1500	2100
SOD (mm)	1.5	3.0	4.5	-
AMFR (g/min)	300	600	-	-

**Table 4:** Constant parameters during the tests.

Orifice Diameter (mm)	Nozzle Diameter (mm)	Nozzle Lenght (mm)	Abrasive Size (µm)	Abrasive Type	Water Pressure (MPa)
0.30	0.8	94.7	120	Garnet	450

**Table 5:** Results obtained during the evaluation for each combination of parameters.

Test	TFR (mm/min)	SOD (mm)	AMFR (g/min)	T (°)	Zone 1 (µm)	Zone 2 (µm)	Zone 3 (µm)
1	300	1.5	300	0.880	5.87	5.03	5.44
2	300	1.5	600	0.761	6.10	4.24	4.27
3	300	3.0	300	1.171	6.08	5.30	6.01
4	300	3.0	600	0.996	6.74	5.15	4.32
5	300	4.5	300	3.457	10.54	6.51	7.58
6	300	4.5	600	3.743	10.70	6.67	6.19
7	900	1.5	300	2.283	7.05	6.31	7.19
8	900	1.5	600	2.517	5.75	5.38	5.53
9	900	3.0	300	2.634	7.50	6.33	6.68
10	900	3.0	600	2.751	6.90	5.48	5.78
11	900	4.5	300	2.926	9.45	7.21	7.79
12	900	4.5	600	3.160	9.35	6.38	6.11
13	1500	1.5	300	3.160	9.64	6.95	9.68
14	1500	1.5	600	3.393	6.71	6.14	6.41
15	1500	3.0	300	3.277	8.61	8.26	9.64
16	1500	3.0	600	3.103	9.18	6.92	7.11
17	1500	4.5	300	4.210	11.68	9.11	11.12
18	1500	4.5	600	4.151	13.45	11.13	9.98
19	2100	1.5	300	2.692	8.71	9.46	10.49
20	2100	1.5	600	3.043	7.90	6.83	7.08
21	2100	3.0	300	3.860	9.88	10.14	10.51
22	2100	3.0	600	4.200	9.29	8.18	8.92
23	2100	4.5	300	4.908	13.98	8.87	9.65
24	2100	4.5	600	5.257	15.36	9.02	7.50

**Table 6:** Analysis of variance (ANOVA) of the evaluated variables.

TFR				SOD				AMFR			
Variable	F-Value	*p*-Value		Variable	F-Value	*p*-Value		Variable	F-Value	*p*-Value	
T	20.18	0.000	**S**	T	21.45	0.000	**S**	T	0.40	0.534	-
Zone 1	16.82	0.000	**S**	Zone 1	52.35	0.000	**S**	Zone 1	0.11	0.744	-
Zone 2	17.65	0.000	**S**	Zone 2	8.36	0.003	**S**	Zone 2	3.26	0.089	-
Zone 3	26.62	0.000	**S**	Zone 3	4.73	0.023	-	Zone 3	31.45	0.000	**S**
